# Training the next generation of pluripotent stem cell researchers

**DOI:** 10.1186/1479-5876-6-40

**Published:** 2008-07-23

**Authors:** Philip Hitchins Schwartz

**Affiliations:** 1 Center for Translational Research, Children's Hospital of Orange County Research Institute, 455 South Main Street, Orange, California 92868 USA; 2 Center for Neuroscience Research, Children's Hospital of Orange County Research Institute, 455 South Main Street, Orange, California 92868 USA

## Abstract

Human pluripotent stem cells (PSCs) have the unique properties of being able to proliferate indefinitely in their undifferentiated state and of being able to differentiate into any somatic cell type. These cells are thus posited to be extremely useful for furthering our understanding of both normal and abnormal human development, providing a human cell preparation that can be used to screen for new reagents or therapeutic agents, and generating large numbers of differentiated cells that can be used for transplantation purposes. PSCs in culture have a specific morphology and they express characteristic surface antigens and nuclear transcription factors; thus, PSC culture is very specific and requires a core skill set for successful propagation of these unique cells. Specialized PSC training courses have been extremely valuable in seeding the scientific community with researchers that possess this skill set.

Human pluripotent stem cells (PSCs) have the unique properties of being able to proliferate indefinitely in their undifferentiated state and of being able to differentiate into any somatic cell type. These cells are thus posited to be extremely useful for furthering our understanding of both normal and abnormal human development, providing a human cell preparation that can be used to screen for new reagents or therapeutic agents, and generating large numbers of differentiated cells that can be used for transplantation purposes [1,2]. PSCs in culture have a specific morphology and they express characteristic surface antigens and nuclear transcription factors; thus, PSC culture is very specific and requires a core skill set for successful propagation of these unique cells. Specialized PSC training courses have been extremely valuable in seeding the scientific community with researchers that possess this skill set.

Most current lines of human PSCs have been obtained from the inner cell mass of embryos produced by in vitro fertilization. These embryo-derived PSCs (ePSCs, more commonly known as ESCs or embryonic stem cells) are currently the "gold standard" for PSCs. A new source of PSCs has recently become available, however: somatic cells that are re-programmed to PSCs – induced PSCs (iPSCs). To generate iPSCs, somatic cells are genetically transduced to express once-silent developmental genes that re-program these cells into PSCs. Two recent publications have demonstrated the success of this technique with human cells. In one case, the cells were transduced to express the transcription factors Nanog, Oct4, Sox2, and Lin28 [3]. In the other, c-Myc, Oct4, Sox2, and Klf4 were required for the re-programming[4]. The iPSCs are of particular importance as they provide a potential source of patient-specific cells for transplantation, i.e. immune-matched cells, as well as the opportunity to study PSCs created from cells of patients with specific genetic diseases. As these cells do not have the ethical baggage associated with ePSCs[5,6], they are likely to be accepted as research tools all over the world.

As with all PSCs, a specific skill set is required for propagation of iPSCs. Moreover, the transformation of a starting pleiomorphic cell in the midst of a dense culture of similar pleiomorphic cells to a very small colony of cells resembling a PSC colony requires not only the appropriate application of genetic reprogramming technology but a trained eye to recognize the morphological progression that the cells undergo. That is, additional skill sets are required for iPSCs: 1) the appropriate lentiviral transduction techniques, using multiple vectors necessary for reprogramming, 2) an ability to recognize the appearance of the re-programmed cells amongst a large excess of non-reprogrammed cells, and 3) an ability to "pick" colonies of appropriately reprogrammed cells and then expand them to derive the new iPSC line and characterize them to prove that they are, in fact, PSCs. Bringing in ePSC biologists and training them in this new technology is but a first step in advancing this field. Another step is to bring in and train scientists outside the PSC field, such as other biologists, chemists, materials scientists, and biomedical engineers, so that entirely new and diverse approaches might be brought to bear on this important area.

Children's Hospital of Orange County (CHOC) just received a one-of-a-kind federal award from the National Institutes of Health (NIH). This award establishes CHOC as having the first federally-funded training grant to incorporate new induced pluripotent stem cell derivation technology as part of its training curriculum. This curriculum is a critical adjunct to the NIH's recent stem cell initiative that will fund new research grants that propose to use this new technology. Dr. Philip H. Schwartz, a Senior Scientist in the Centers of Neuroscience and Translational Research at the CHOC Research Institute and the principal investigator of the grant, will teach this new technology which allows the derivation of pluripotent stem cells from adult cells, rather than from human embryos.

CHOC has been running 10-day, intensive, PSC culture training courses since 2004, being one of the first in the country and the first in the Western United States to continuously offer this comprehensive, specialized training. These NIH-sponsored training courses provide hands-on training for investigators and, by bringing together some of the leading experts on PSC technology, train students in the successful culture, maintenance, and manipulation of ePSCs. In addition, the course directors have published very well-received book, Human Stem Cell Culture – A Laboratory Guide[7], that details all of the technologies that the course covers, as well as other techniques relevant to the field. With the additional NIH funding, students of the next course can now be taught iPSC derivation, isolation, and propagation, in addition to the now-standard ePSC techniques. iPSCs will be derived from fibroblasts, using existing methodologies, by transducing them with lentiviral vectors encoding for the transcription factor genes OCT4, NANOG, LIN28, and SOX2. Students will be taught how to isolate and characterize iPSCs and will also be taught proper techniques for banking PSCs. All of these techniques will be taught using a temporal shift methodology such that students, at the very beginning of the course, will be given cultures at all major stages, from fibroblast, to very early re-programming, to establishment of colonies, to expanding colonies, to colonies ready for banking. The students will take these cultures and advance each of them to the next stage and characterize them appropriately. Thus, by the end of the 10-day course, the students will have been well-exposed, in compressed temporal sequence, to what takes many weeks to accomplish in real practice and, thus, will possess the essential skill set needed for properly deriving and characterizing iPSCs in their own laboratories.

Dr. Schwartz' stem cell research laboratory at CHOC focuses on the use of stem cells to understand the neurobiological causes of autism and other neurodevelopmental disorders such that new treatments may be discovered. Preclinical trials using stem cell therapy in a model of a lysosomal storage disease are also in progress in his laboratory. Finally, the laboratory houses the National Human Neural Stem Cell Resource , a repository of human stem cells that are made widely available to the scientific community. The on-line application for the training course is found at this site. Dr. Schwartz welcomes e-mail at pschwartz@choc.org.
